# Cost–Utility and Budget Impact Analyses of Oral Chemotherapy for Stage III Colorectal Cancer: Real-World Evidence after Policy Implementation in Thailand

**DOI:** 10.3390/cancers15204930

**Published:** 2023-10-11

**Authors:** Pochamana Phisalprapa, Chayanis Kositamongkol, Krittiya Korphaisarn, Charuwan Akewanlop, Vichien Srimuninnimit, Siripen Supakankunti, Natnasak Apiraksattayakul, Nathorn Chaiyakunapruk

**Affiliations:** 1 Division of Ambulatory Medicine, Department of Medicine, Faculty of Medicine Siriraj Hospital, Mahidol University, Bangkok 10700, Thailand; 2 Division of Medical Oncology, Department of Medicine, Faculty of Medicine Siriraj Hospital, Mahidol University, Bangkok 10700, Thailand; 3 Centre of Excellence for Health Economics, Faculty of Economics, Chulalongkorn University, Bangkok 10330, Thailand; 4 Faculty of Medicine Siriraj Hospital, Mahidol University, Bangkok 10700, Thailand; 5 College of Pharmacy, University of Utah, Salt Lake City, UT 84112, USA; 6 IDEAS Center, Veterans Affairs Salt Lake City Healthcare System, Salt Lake City, UT 84108, USA

**Keywords:** budget impact analysis, capecitabine, colorectal cancer stage III, cost–utility analysis, oral chemotherapy

## Abstract

**Simple Summary:**

There are various chemotherapy regimens used to treat patients diagnosed with stage III colorectal cancer, one of which is an oral chemotherapy drug called “capecitabine”. Our study examined the cost-effectiveness of eight chemotherapy regimens using a Markov model. The analysis was performed from a societal perspective with a lifetime time horizon. The results demonstrated that the most cost-effective strategy for treating patients is to begin with a chemotherapy regimen that includes capecitabine and oxaliplatin and then add irinotecan if the disease progresses. However, the budget impact of this strategy was estimated to be approximately USD 25.1 million, which is about three times higher than the regimen that involves only 5-fluorouracil/leucovorin and oxaliplatin. Policymakers should consider the relatively high budgetary burden of the regimen.

**Abstract:**

This study conducted a cost–utility analysis and a budget impact analysis (BIA) of outpatient oral chemotherapy versus inpatient intravenous chemotherapy for stage III colorectal cancer (CRC) in Thailand. A Markov model was constructed to estimate the lifetime cost and health outcomes based on a societal perspective. Eight chemotherapy strategies were compared. Clinical and cost data on adjuvant chemotherapy were collected from the medical records of 1747 patients at Siriraj Hospital, Thailand. The cost-effectiveness results were interpreted against a Thai willingness-to-pay threshold of USD 5003/quality-adjusted life year (QALY) gained. A 5-year BIA was performed. Of the eight strategies, CAPOX then FOLFIRI yielded the highest life-year and QALY gains. Its total lifetime cost was also the highest. An incremental cost-effectiveness ratio of CAPOX then FOLFIRI compared to 5FU/LV then FOLFOX, a commonly used regimen USD was 4258 per QALY gained.The BIA showed that when generic drug prices were applied, 5-FU/LV then FOLFOX had the smallest budgetary impact (USD 9.1 million). CAPOX then FOLFIRI required an approximately three times higher budgetary level (USD 25.1 million). CAPOX then FOLFIRI is the best option. It is cost-effective compared with 5-FU/LV then FOLFOX. However, policymakers should consider the relatively high budgetary burden of the CAPOX then FOLFIRI regimen.

## 1. Introduction

Cancer is a major global health problem. An upwards trend in new cancer cases has been predicted, particularly in low-income countries, with the number of new cases worldwide expected to reach 20 million by 2025 [1]. According to the most recent data, colorectal cancer (CRC) is the third most commonly diagnosed cancer globally, and its mortality rate ranks second among all cancer types [2]. Approximately 1.9 million new cases of CRC and 900,000 resulting deaths occurred in 2020 [2]. The incidence of CRC is higher in more-developed regions, but its mortality is higher in less developed areas.

In Thailand, CRC was reported to be the third and fourth leading cancer in men and women, respectively. The mean annual age-standardized incidence rates per 100,000 of the population were 16.2 in men and 11.1 in women [3]. CRC was detected much more frequently in the population aged between 50 and 75 years. The proportion of Thai patients with CRC who were diagnosed with stage III cancer varied from 16% to 71%, depending on where the survey was conducted [4].

Adjuvant chemotherapy in stage III CRC is required to prolong disease-free and overall survival. It is recommended as standard treatment by both international and local CRC treatment guidelines [4,5]. The 5-year disease-free survival of patients with stage III CRC who received adjuvant chemotherapy was approximately 64% (95% confidence interval [CI]: 59.3–67.9) [4], compared with 49% (95% CI: 23.2–74.8) in those who did not receive chemotherapy. However, various orally and intravenously administered chemotherapy agents are available. Orally administered chemotherapy for stage III CRC includes capecitabine, while intravenously administered chemotherapy includes 5-fluorouracil/leucovorin (5-FU/LV) and oxaliplatin. The various chemotherapy regimens have demonstrated different efficacy and toxicity profiles. Published evidence has confirmed that capecitabine and oxaliplatin show better efficacy than 5-FU/LV monotherapy [6,7,8,9]. In addition, the preference of patients and the treatment costs of capecitabine and oxaliplatin versus FU/LV monotherapy are different [9]. Capecitabine has been approved by the Thai Food and Drug Administration. However, its price has been relatively high compared with 5-FU/LV, because only the original brand of capecitabine was available until recently. With the launch of generic versions of capecitabine in the Thai pharmaceutical market, treatment costs have become much lower.

There have been several studies on the pharmacoeconomics of adjuvant chemotherapy. Most concluded that capecitabine or 5-FU/LV in combination with oxaliplatin (CAPOX or FOLFOX) was more effective than 5-FU/LV alone [10,11,12,13,14,15]. Regarding Thai survey data, patients treated with an orally administered agent reported more convenience than those treated with intravenous regimens [16]. Current data from Thailand indicate that the overall annual hospital charge to patients with CRC is over USD 54 million, with an average charge per admission per patient of USD 1283. The average hospital charges per admission were USD 2008, USD 1547, and USD 893 for government welfare, social welfare, and universal health coverage schemes, respectively. The average hospital charges vary among insurance schemes because of differences in the drug accessibility of each scheme [17]. In 2020, capecitabine and irinotecan were listed in the National List of Essential Medicines of Thailand for stage III and progressive CRC, respectively. Their listing made the drugs much more accessible to patients with CRC.

Research data have also shown an increasing CRC burden in low- and middle-income countries, whereas stabilizing or decreasing trends were identified only in highly developed countries. Thus, a sustainable policy of better management options is necessary for patients with CRC living in less developed areas [18]. The best available chemotherapy regimens for CRC should be accessible to all patients without causing an economic crisis in the national public health system. A successful national policy should deliberate on clinical outcomes, patients’ health-related quality of life, and the country’s limited resources. The policy also requires long-term planning and post-policy implementation re-evaluation.

This study conducted a cost–utility analysis and a budget impact analysis (BIA) of outpatient oral chemotherapy versus conventional intravenous chemotherapy for stage III CRC in Thailand.

## 2. Methods

### 2.1. Overall Description

A cost–utility analysis was performed to estimate the related costs and health outcomes of patients with stage III CRC. Four chemotherapy agents—5-FU/LV, capecitabine, oxaliplatin, and irinotecan—were combined to form 8 treatment strategies, each composed of 2 chemotherapy regimens. The first regimen was adjuvant chemotherapy for stage III CRC, while the second was for patients with a recurrence or a progressive stage (stage IV or metastatic CRC). The 8 treatment strategies were as follows: (1) 5-FU/LV then FOLFOX, (2) 5-FU/LV then CAPOX (capecitabine in combination with oxaliplatin), (3) 5-FU/LV then FOLFIRI (5-FU/LV in combination with irinotecan), (4) capecitabine then FOLFOX, (5) capecitabine then CAPOX, (6) capecitabine then FOLFIRI, (7) FOLFOX then FOLFIRI, and (8) CAPOX then FOLFIRI. All 8 strategies were included in our analysis (Table 1).

Per the recommendations of the Thai Health Technology Assessment Guidelines (version 2.0) [19], a societal perspective and a lifetime horizon were incorporated into the cost–utility analysis. Future costs and outcomes were discounted at 3% per annum [19]. The results were reported in terms of incremental costs, life years (LYs) gained, and quality-adjusted life years (QALYs) gained. Each compared strategy’s incremental cost-effectiveness ratio (ICER) was also calculated. In the case of a positive ICER, a ceiling threshold of cost-effectiveness was set at USD 5003 per QALY gained, representing the maximum value of the Thai social willingness to pay (WTP) indicated in the Thai Health Technology Assessment Guidelines [19,20]. The Siriraj Institutional Review Board approved the study protocol (376/2017).

### 2.2. Economic Model

**Figure 1:**
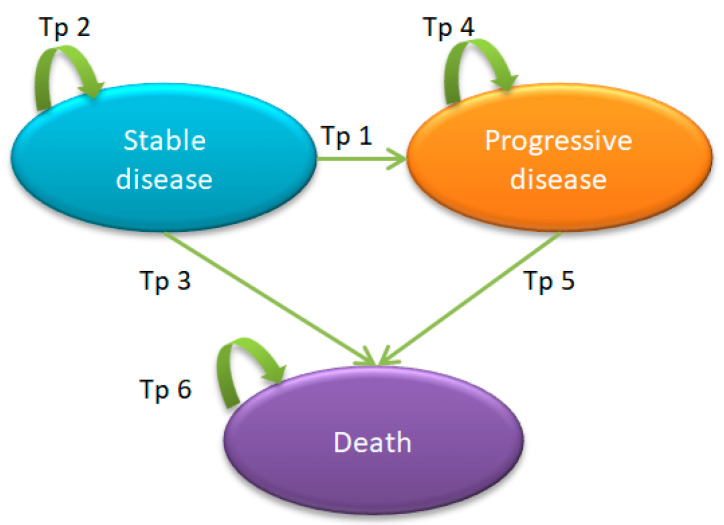
A Markov model describing the disease progression in stage III colorectal cancer.

A Markov model with a 1-year cycle length was employed to capture the long-term outcomes of each treatment strategy. The model consisted of 3 hypothetical health stages: stable disease, progressive disease, and death [16], as shown in Figure 1. At the beginning of the analysis, all hypothetical patients were assumed to be newly diagnosed with stage III CRC. We hypothesized that the disease could progress to the adjacent stage (i.e., progressive disease) or death in the next cycle. The patients could also remain in the same stage but could not move to any previous health stage. All of the hypothetical patients were followed until death. We assumed a patient age of 63 years when entering patient details into the Markov model. This is the average age of Thai patients at the time of their diagnosis with stage III CRC (unpublished data from Siriraj Hospital’s electronic database; *n* = 951).

### 2.3. Model Input Parameters

The model input parameters drew upon data on Thai patients with CRC reported in the literature or held in the electronic database of the Siriraj Colorectal Cancer Registry. This registry is maintained at Siriraj Hospital, which is the largest tertiary care center in Thailand. The primary data used in the model were calculated and summarized from 1747 patients who had received adjuvant chemotherapy at Siriraj Hospital from 2009 to 2014. Of those patients, 951 had stage III CRC, whereas the remaining 796 patients had stage IV CRC. All patients were monitored until death or for at least 5 years. The model input parameters are listed in Table 2.

The demographic and clinical data of patients in Siriraj Colorectal Cancer Registry were described as follows. The mean age at diagnosis of 951 patients with stage III CRC was 63.0 ± 12.9 years. About half of these cohort were men (54.3%). Stage III colon cancer was diagnosed in 47.5% of the patients; the remaining patients were diagnosed with rectal cancer. The proportions of first chemotherapy regimen after the diagnosis were 38.2%, 24.9%, 11.4%, and 25.5% for 5-FU/LV, capecitabine, FOLFOX, and CAPOX, respectively. The total follow-up time was 3722 patient years. Patients with stage IV CRC in the registry had the mean age at diagnosis of 61.9 ± 12.5 years. Fifty-seven percent of these were men. Colon cancer was diagnosed in 52.4% of patients with stage IV CRC; the rest were diagnosed with rectal cancer. Initial chemotherapy regimens prescribed after the diagnosis included FOLFOX, CAPOX, and FOLFIRI, with proportions of 30.0%, 66.2%, and 3.8%, respectively. The total follow-up time of patients with a first diagnosis of stage IV CRC was 1432 patient years.

#### 2.3.1. Treatment Options and Effectiveness

Under current CRC treatment practices in Thailand, patients with stage III CRC are primarily treated with 4 chemotherapy regimens: 5-FU/LV, capecitabine, FOLFOX, and CAPOX. In our Markov model, the effectiveness of each regimen was reflected in the transition probabilities from disease-free to disease recurrence and, ultimately, to death. Once the disease progresses, 3 regimens for stage IV CRC are available: FOLFOX, CAPOX, and FOLFIRI. The scope of this study did not extend to other possible treatments, such as immunotherapy or targeted therapy. The dose of each chemotherapy agent was calculated based on an average Thai population body surface area of 1.7 m^2^ (body weight: 60 kg), as recommended by the Thai CRC treatment guidelines.

#### 2.3.2. Probability Data

The probability data were derived from the Siriraj Colorectal Cancer Registry database (unpublished data; *n* = 1747) and work by Lerdkiattikorn et al. [16]. The transition probabilities to death were considered all-cause mortality; they varied depending on the disease status, patient age, and amount of time spent at the same state. The probabilities of death were calculated by combining the age-specific mortality rate (adopted from the World Health Organization Life Table 2019 [24]) and the disease-specific mortality rate of patients with stage III and IV CRC (obtained from the Siriraj Colorectal Cancer Registry).

#### 2.3.3. Cost Data

As we focused on the societal perspective, the cost–utility analysis included 2 types of costs: direct medication and direct non-medical costs. However, indirect costs were not included. This was because any impaired ability to work or to engage in leisure activities resulting from morbidity was captured by a decrease in the QALY value [19]. All cost data were local costs that were converted into USD as of 2021. Drug costs were obtained from the National Drug Reference Price database of the Drug and Medical Supply Information Center, Ministry of Public Health [22]. Our analysis used the median of the median reference price of generic drugs., as recommended by the Thai Health Technology Assessment Guidelines [19]. The National List of Essential Medicines price was applied where no data were available. We assumed no product wastage in our analysis. Consequently, the total chemotherapy cost per recommended dose was calculated from the net cost per milligram of a drug multiplied by the milligrams required per dose (Table 1).

Other healthcare costs were obtained from Siriraj Hospital’s electronic database. These costs were related to surgical treatment, intravenous drug administration, outpatient department follow-ups, inpatient department visits due to worsening disease status, and adverse event treatment costs. Inpatient department visits for drug administration and direct non-medical costs (food and transportation) were obtained from the reference prices published in the Thai Standard Cost Lists for Health Technology Assessments [23]. Costs from Siriraj Hospital’s database were elicited with the help of the Information Technology Department. A total of 1747 patients with CRC were included in the cost analysis (951 cases with stage III CRC and 796 cases with stage IV CRC). We separated the costs of the follow-ups into 3 periods (the first, second, and third years after diagnosis) because we were concerned about the differences in the average costs per visit and the hospital visit rate of each year after the CRC diagnosis. The costs of colonoscopies and their associated complications were already included in the costs from Siriraj Hospital’s database.

All costs were converted to USD as of 2021 using an exchange rate of THB 31.98 per USD 1 [25] and the Thai consumer price index [26]. The cost details are shown in Table 2.

#### 2.3.4. Utility Data

We adopted utility data from a study by Lerdkiattikorn et al., who carried out a survey using the EQ-5D questionnaire to estimate the utilities of Thai patients with CRC in different disease states. Their results also showed the difference in utility scores of patients who received chemotherapies that required different administration routes (Table 2).

### 2.4. Cost–Utility Analysis

#### 2.4.1. Base Case Analysis

We compared the total lifetime costs and health outcomes of each treatment strategy using 5-FU/LV then FOLFOX as the base case.

#### 2.4.2. Sensitivity Analyses

One-way sensitivity analysis was employed to evaluate the impact of uncertainty in the model input parameters. Clinical effects and utilities were varied within the range of 95% CI and costs were varied within ±25% from the base case values to determine their impacts on the ICER. The results are presented as a tornado diagram. The one-way sensitivity analyses were only conducted for treatment strategies that demonstrated the highest lifetime QALY gain compared with the base case strategy.

Probabilistic sensitivity analyses were performed using 1000 iterations of a Monte Carlo simulation. The results of the probabilistic sensitivity analyses are presented in a cost-effectiveness plane and as cost-effectiveness acceptability curves. Transition probabilities and utilities were assumed to follow a beta distribution, while cost data were assumed to have a gamma distribution. The expected net monetary benefit was calculated for the range of the WTP thresholds from USD 0 to USD 20,000 per QALY gained to show the probability of being the best buy option compared with the base case strategy.

#### 2.4.3. Budget Impact Analysis

A BIA of the base case strategy (5-FU/LV then FOLFOX) and other possible strategies of chemotherapy treatments were performed to estimate the budget impact for the next 5 years and to determine the differences in the budgetary needs of each strategy. The total population, the incidence rate of stage III CRC, and the relapse rate of the first regimen for treating stage III CRC were used to calculate the number of patients requiring treatment. The most up-to-date median drug prices were used in the BIA. The analysis was conducted from a payer’s perspective: we included only drug prices and their administration costs. The budgetary impact was calculated at an 80% accessibility rate (i.e., 80% of newly diagnosed stage III CRC patients and 80% of those whose disease relapsed). The other 20% of patients with CRC were handled by palliative care. The 80% accessibility rate was based on data from the Siriraj Colorectal Cancer Registry. The population growth rate was assumed to be 0.3% per annum [21]. The parameters used in the BIA are detailed in Table 2.

## 3. Results

### 3.1. Base Case Analysis

Among the eight adjuvant chemotherapy strategies, CAPOX then FOLFIRI provided the highest number of total lifetime QALYs of 3.60, with 6.87 years of life expectancy. On the other hand, 5-FU/LV then FOLFIRI provided the lowest number of QALYs per lifetime, with a life expectancy of 4.52 years. The most expensive strategy was FOLFOX then FOLFIRI, which generated a total of USD 26,259 in costs throughout a patient’s lifetime. The base case strategy (5FU/LV then FOLFOX) was not the strategy with the lowest cost: its total lifetime cost was USD 17,092. Unfortunately, among the eight strategies, it provided the second fewest years of life expectancy, with 2.54 QALYs. When compared other strategies against the base case, both 5-FU/LV then CAPOX and capecitabine then CAPOX were considered cost-saving strategies; additionally, CAPOX then FOLFIRI was considered cost-effective. The remaining chemotherapy treatment strategies (5-FU/LV then FOLFIRI, capecitabine then FOLFIRI, FOLFOX then FOLFIRI, and capecitabine then FOLFOX) were not cost-effective in Thailand’s context. Details are provided in Table 3.

### 3.2. Sensitivity Analyses

#### 3.2.1. One-Way Sensitivity Analyses

**Figure 2:**
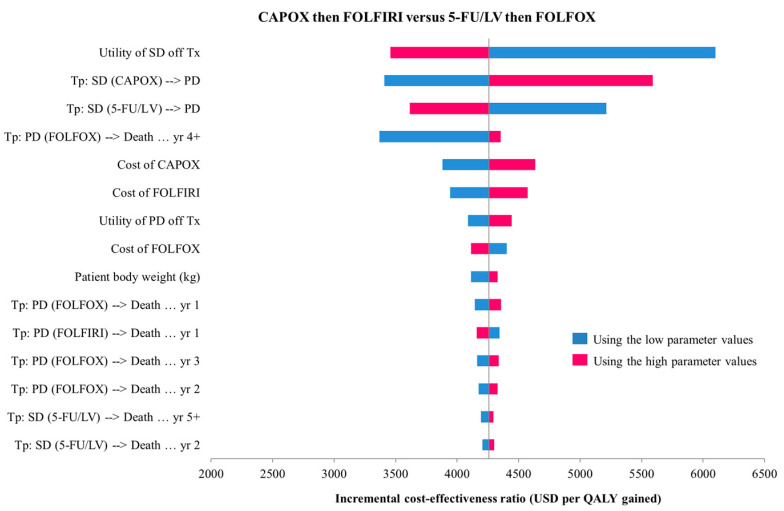
Tornado diagram of treatments with CAPOX then FOLFIRI compared with 5-FU/LV then FOLFOX. PD, progressive disease; SD, stable disease; Tx, treatment; Tp, transition probability; yr, year.

Figure 2 shows a tornado diagram illustrating the results of the one-way sensitivity analyses of CAPOX then FOLFIRI versus 5-FU/LV then FOLFOX. Only the top 15 influencing parameters were included in the diagram. In descending order of sensitivity, the ICER was most sensitive to the utility of stable disease when off treatment, to the transition probability of CAPOX from stable disease to progressive disease, to the transition probability of 5-FU/LV from stable disease to progressive disease, to the transition probability of FOLFOX from progressive disease to death in the fourth and subsequent years, and to the cost of CAPOX.

We found that the ICER rose above the Thai WTP threshold when we applied the following:

- the lower-limit values of the utility of stable disease when off treatment;
- the upper-limit value of the transition probability of CAPOX from stable disease to progressive disease;
- the lower-limit values of the transition probability of 5-FU/LV from stable disease to progressive disease, with respect to the descending order of sensitivity.

#### 3.2.2. Probabilistic Sensitivity Analyses

**Figure 3:**
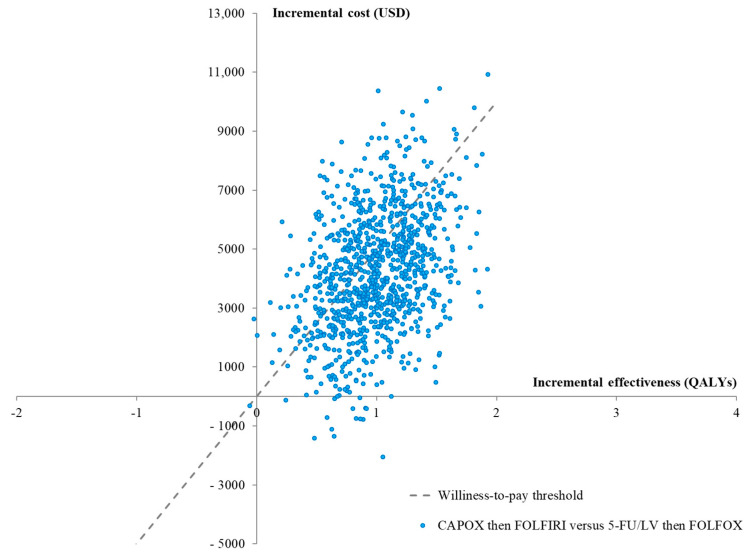
Cost-effectiveness plane of CAPOX then FOLFIRI versus 5-FU/LV then FOLFOX.

**Figure 4:**
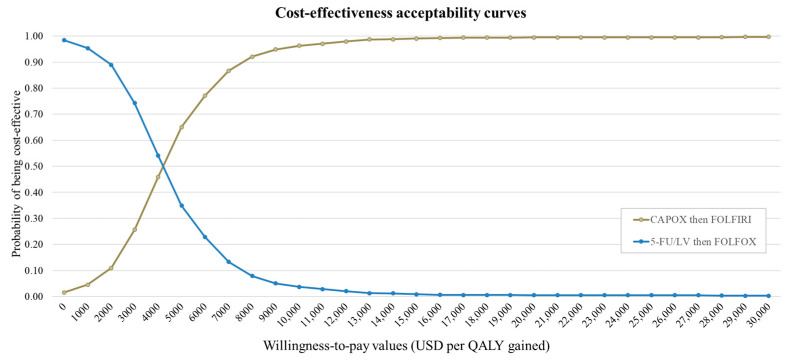
Cost-effectiveness acceptability curve of CAPOX then FOLFIRI versus 5-FU/LV then FOLFOX.

The results of the probabilistic sensitivity analyses are presented in a cost-effectiveness plane (Figure 3) and as cost-effectiveness acceptability curves (Figure 4). The curves show the superiority of CAPOX then FOLFIRI over 5-FU/LV then FOLFOX at approximately WTP values of above USD 4500 per QALY gained. At the current Thai WTP threshold, the probability of CAPOX then FOLFIRI being cost-effective was 65.2%, compared with the base case.

### 3.3. Budget Impact Analysis

The 5-year time horizon BIA showed that at the current median values of generic drug prices in Thailand, 5-FU/LV then FOLFOX had the smallest budgetary impact (about USD 9.1 million). In contrast, CAPOX then FOLFIRI ranked as the strategy with the second highest budgetary impact. Its budget impact was USD 25.1 million, about three times higher than that of the base case strategy). Details are provided in Table 4.

## 4. Discussion

Oral chemotherapy was more favorable than intravenous chemotherapy in terms of convenience. The Siriraj Colorectal Cancer Registry data indicated that the average 5-year mortality rates of 5-FU/LV, capecitabine, FOLFOX, and CAPOX in stage III CRC were 0.13, 0.08, 0.05, and 0.04 deaths per year, respectively. The average 4-year mortality rates of FOLFOX, CAPOX, and FOLFIRI in stage IV CRC were 0.40, 0.35, and 0.69 deaths per year. Capecitabine-based regimens involved lower administration and direct non-medical costs from a societal perspective. An oral drug delivery system could reduce the monetary burdens on patients and healthcare systems. Additionally, the workload of healthcare professionals would decrease if orally administered chemotherapy were set as a standard treatment in routine practice. This is because an oral drug delivery system would abate the number of hospital visits and admissions resulting from intravenous drug administrations and their side effects.

This study is the third economic evaluation of CRC treatment in Thailand. Our findings are similar to the first and second cost–utility studies by Lerdkiattikorn et al. in 2014 [16] and Katanyoo et al. in 2018 [27]. Those two studies found that oral chemotherapy provided higher lifetime QALYs than intravenous chemotherapies, but it was also more expensive [16,27]. Furthermore, the studies concluded that their most effective regimens (Lerdkiattikorn et al.: a first-line FOLFOX then FOLFIRI; Katanyoo et al.: capecitabine) were not cost-effective at the Thai WTP threshold compared with their base cases (Lerdkiattikorn et al.: a first-line 5-FU/LV then capecitabine; Katanyoo et al.: a 5-FU/LV regimen).

In contrast, our study conducted analyses using 5-FU/LV then FOLFOX as the base case strategy instead of 5-FU/LV then capecitabine. We employed this base case as it aligned with the medical practice at the time, when capecitabine and oxaliplatin had not yet been listed in the National List of Essential Medicines for stage III CRC treatment, and there is evidence that FOLFOX provides significantly better efficacy than capecitabine in patients with relapsed CRC. The total lifetime costs from the previous models were much higher than our results, primarily because of differences in drug prices. The prices of chemotherapy agents have significantly dropped due to the availability of generic versions in the market. However, the other healthcare costs we found were similar to those of the two earlier investigations [16,27]. Our post-policy implementation analysis showed that CAPOX then FOLFIRI was a cost-effective strategy compared with the base case at the current median drug prices. Capecitabine’s price has fallen by approximately 60% from the value applied in the two prior economic evaluations of chemotherapy in CRC, i.e., the price in force in 2013 [16,27].

According to the present study, CAPOX then FOLFIRI provided the highest number of QALYs, with a lower total lifetime cost than FOLFOX then FOLFIRI. These results differed from those of Lerdkiattikorn et al. [16]. They reported that FOLFOX then FOLFIRI provided the most QALYs gained and the lowest ICER compared with the base case of 5-FU/LV then capecitabine. This discordance arose because we drew upon different sources for the input parameters. Our parameters were up-to-date and more specific to Thai patients as they were mainly extracted from the data on the 1747 patients with stage III and stage IV CRC in the Siriraj Colorectal Cancer Registry. FOLFIRI is an effective treatment for advanced-stage CRC, according to international guidelines [28]. For patients previously treated with an oxaliplatin-based regimen, irinotecan is the only reasonable and effective choice when the disease relapses. This is particularly the case for patients who cannot afford more costly medicines, such as patients who are part of Thailand’s Universal Health Coverage Scheme. Irinotecan is much cheaper than other targeted therapies or immunotherapy. Both capecitabine and irinotecan are presently included in the National List of Essential Medicines of Thailand. Capecitabine is available for patients with stage III CRC, and irinotecan is listed for patients with progressive disease. These drugs should be accessible to all patients with indications unless they cannot tolerate them due to their poor health condition or their decision not to be treated by these regimens. Another important note for policymakers is that capecitabine might not be appropriate for patients with severe renal impairment. Thus, national policymakers should recognize that various stage III CRC treatment options are still needed.

The sensitivity analyses showed that when the CAPOX then FOLFIRI regime was compared against 5-FU/LV then FOLFOX, changes in some utility values and transition probabilities increased its ICER above the Thai WTP threshold. CAPOX then FOLFIRI showed a higher probability of being cost-effective than 5-FU/LV then FOLFOX, given the WTP threshold of approximately USD 4500 per QALY gained. This threshold is lower than current WTP of Thailand.

Regardless of the availability of targeted therapy and immunotherapy, CAPOX then FOLFIRI is the most recommended strategy because it has provided good clinical outcomes according to the guidelines [28]. However, despite capecitabine and irinotecan being listed in the National List of Essential Medicines of Thailand, in terms of the budget impact analysis from the payer’s perspective, its cost is about three times that of 5-FU/LV then FOLFOX. Its budget impact should be able to reduce if the prices of capecitabine and irinotecan continue to decline in the highly competitive generic medicine market. 

Several economic evaluations of chemotherapy for stage III CRC have been published previously in both developed and developing countries. In a high-income setting, such as the United Kingdom, the X-ACT trial [11,29,30] showed that despite the higher cost of oral capecitabine, it was associated with lower adverse events and societal costs (i.e., travel and time costs). This resulted in a lower total lifetime cost for the treatment, compared to its intravenous form. The study concluded that capecitabine should be used for stage III CRC treatment instead of 5-FU/LV.

With regard to Asia, a cost-effectiveness study in Japan reported that when compared to 5-FU/LV, capecitabine was associated with lower direct medical cost, despite providing more QALYs [31]. This made capecitabine-based treatment a dominant strategy. A Taiwanese study examined the economic costs associated with capecitabine-based and 5-FU/LV-based adjuvant treatments for older adults with stage III CRC. The study concluded that the adjusted monthly treatment cost of the capecitabine-based regimen was significantly lower than that of the 5-FU/LV-based regimen (USD 978.5 versus USD 1923.7, respectively, at a conversion rate of USD 1 = TWD 27.9) [32].

In a lower income area, as in South Africa, CAPOX has been identified as an optimally cost-effective regimen. Its total lifetime cost was found to be the lowest (international dollar 5381), with 5.74 disability-adjusted life years averted [33]. Their simulation indicated that FOLFOX provided the highest number of disability-adjusted life year averted when compared with 5-FU/LV, capecitabine, and CAPOX. However, because of its greater total cost, its ICER exceeded the WTP of South Africa.

This study has notable strengths. First, it used model input parameters based on data from the Siriraj Colorectal Cancer Registry and real-world local evidence. Consequently, the results were highly specific to Thai patients. In addition, the mortality rate applied in our model was adjusted using the Thai population’s age-specific mortality rate. The use of local data as much as possible in our analyses might be beneficial in evaluating the success of the current national policies for stage III CRC treatment. Second, gastroenterologists and health economists were involved throughout the cost–utility analysis and BIA. This approach ensured the validity of the model inputs and the interpretations of the results. Low- and middle-income countries can use the data from our Thai study to demonstrate the value of CRC treatment. Our findings will also help to focus clinicians’ and policymakers’ attention on the cost-effectiveness and budgetary impact of various stage III CRC treatment options. This is needed because the disease has become a global burden, and its incidence is rapidly rising. Finally, this is the first study of post-policy implementation analysis in Thailand.

However, the study has some limitations. One is that our analysis did not cover immunotherapy or targeted therapy for the treatment of stage IV CRC due to their lack of affordability. These medications have not yet been included in the National List of Essential Medicines of Thailand. Furthermore, we intended to specifically focus the study on the results of policy implementation in low- and middle-income countries that have universal health coverage systems. Limited access to high-cost drugs is an issue in these countries. However, if future clinic research confirms that immunotherapy and targeted therapies, as well as other novel forms of chemotherapy, have better efficacy than current chemotherapy regimens and acceptable safety profiles and become more affordable, updated cost–utility analyses and BIAs should be performed.

Nowadays, development is ongoing regarding drug delivery systems which intend to improve the quality of cancer treatments. For example, nanosized drug delivery systems are a promising innovative strategy aimed to increase the efficacy and reduce the toxicity of conventional agents [34]. However, upcoming technology will inevitably be accompanied with higher acquisition costs. Whenever these novel technologies become available, they should be included in economic evaluation research because their outcomes will provide important evidence for resource management and the policy development process. On the other hand, clinical trials are still needed to ensure the efficacy and safety of the agents.

Currently, combination regimens of 5-FU/LV or capecitabine with oxaliplatin (i.e., FOLFOX or CAPOX) are now recommended as the standard adjuvant chemotherapy for stage III CRC in Thailand. This recommendation recognizes the well-established superior efficacies of the combination regimens to 5-FU/LV or capecitabine monotherapy. Nevertheless, 5-FU/LV then FOLFOX was chosen as the base case for our analysis because it was a standard strategy in the past when capecitabine and oxaliplatin were not listed in the National List of Essential Medicines of Thailand as the choices of treatment for stage III CRC.

Another limitation is that we could not separate the cost of comorbidity care from the total cost of CRC-related treatment. Consequently, we were obligated to include comorbidity care in the analyses, which could slightly overestimate the CRC treatment costs. A further limitation relates to the data from the Siriraj Colorectal Cancer Registry. We could not extract the mortality data specific to the first-line treatment of patients with progressive disease. Thus, we applied the same mortality rate in patients with the progressive stage as those with stage IV CRC who were naive to chemotherapy. 

## 5. Conclusions

Treatment regimens administered via an oral or outpatient intravenous route are potentially cost-effective strategies for treating stage III CRC in Thailand. This post-policy implementation analysis, using real-word data, emphasizes that CAPOX then FOLFIRI provides the highest life year and QALY gains and is considered cost-effective when compared with 5-FU/LV then FOLFOX. We support the current use of the CAPOX then FOLFIRI strategy as a standard of care for stage III CRC. However, policymakers should consider the relatively high budgetary burden of the CAPOX then FOLFIRI strategy. Its use as a standard treatment for stage III CRC may affect policy sustainability in the future as this particular strategy requires a high budgetary commitment.

## Figures and Tables

**Table 1:** Chemotherapy dosage regimens.

Chemotherapy	Dosage Regimen	Reference
5-FU/LV	- Leucovorin 20 mg/m^2^/day IV bolus, days 1–5 - 5-FU 400 mg/m^2^/day IV bolus after leucovorin, days 1–5 - Repeat every 4 weeks for 6 cycles	[5]
Capecitabine	- Capecitabine 2000 mg/m^2^/day divided into 2 doses, days 1–14, followed by 7 days of rest - Repeat every 3 weeks for 8 cycles	[5]
FOLFOX	- Oxaliplatin 85 mg/m^2^/day IV infusion over 2 h, day 1 - Simultaneously, leucovorin 400 mg/m^2^/day IV infusion over 2 h, day 1 - 5-FU 400 mg/m^2^/day IV bolus day 1, then 2400 mg/m^2^ IV continuous infusion over 46 h. - Repeat every 2 weeks for 12 cycles	[5]
CAPOX	- Capecitabine 2000 mg/m^2^/day PO divided into 2 doses, days 1–14, followed by 7 days of rest - Oxaliplatin 130 mg/m^2^ IV infusion over 2 h, day 1 - Repeat every 3 weeks for 8 cycles	[5]
FOLFIRI	- Irinotecan 180 mg/m^2^ IV infusion over 90 min, day 1 - Leucovorin 400 mg/m^2^ IV infusion over 2 h during irinotecan infusion, day 1 - 5-FU 400 mg/m^2^ IV bolus, then 2400 mg/m^2^ IV continuous infusion over 46 h. - Repeat every 2 weeks for 12 cycles	[5]

IV, intravenous; PO, per oral.

**Table 2:** Model input parameters.

Input Parameters	Distribution	Base Case Values (Standard Error)	Reference
Time horizon		lifetime	[19]
Cycle length (year)		1	
Annual discount rate (range)		3% (0–6%)	[19]
Age-specific incidence rate of stage III CRC per 100,000 population		39.72	[4]
%Eligible case		80%	Primary data
Population growth rate		0.3%	[21]
Patient body weight (kg)		60	Primary data
Patient body surface area (m^2^)		1.7	Mosteller’s formula
***Annual transition probabilities***			
5-FU/LV			
SD to PD	Beta	0.175 (0.012)	[16]
SD to death year 1		0.053	Primary data
SD to death year 2		0.152	Primary data
SD to death year 3		0.199	Primary data
SD to death year 4		0.108	Primary data
SD to death subsequent years		0.121	Primary data
Capecitabine			
SD to PD	Beta	0.149 (0.010)	[16]
SD to death year 1		0.057	Primary data
SD to death year 2		0.073	Primary data
SD to death year 3		0.108	Primary data
SD to death year 4		0.105	Primary data
SD to death subsequent years		0.072	Primary data
FOLFOX			
SD to PD	Beta	0.133 (0.009)	[16]
SD to death year 1		0.000	Primary data
SD to death year 2		0.082	Primary data
SD to death year 3		0.060	Primary data
SD to death year 4		0.063	Primary data
SD to death subsequent years		0.068	Primary data
PD to death year 1		0.208	Primary data
PD to death year 2		0.351	Primary data
PD to death year 3		0.514	Primary data
PD to death subsequent years		0.222	Primary data
CAPOX			
SD to PD	Beta	0.140 (0.010)	[16]
SD to death year 1		0.012	Primary data
SD to death year 2		0.037	Primary data
SD to death year 3		0.071	Primary data
SD to death year 4		0.069	Primary data
SD to death subsequent years		0.030	Primary data
PD to death year 1		0.176	Primary data
PD to death year 2		0.435	Primary data
PD to death year 3		0.318	Primary data
PD to death subsequent years		0.267	Primary data
FOLFIRI			
PD to death year 1		0.474	Primary data
PD to death year 2		0.574	Primary data
PD to death year 3		0.462	Primary data
PD to death subsequent years		0.500	Primary data
***Costs (2021; USD)***			
*Direct medical costs*			
Cost of chemotherapy (2021; USD per dosage unit)			
5-FU (1000 mg/vial)	Gamma	4	[22]
LV (300 mg/vial)	Gamma	9	[22]
Capecitabine (500 mg/tab)	Gamma	2	[22]
Oxaliplatin (100 mg/vial)	Gamma	39	[22]
Irinotecan (100 mg/vial)	Gamma	43	[22]
Cost of chemotherapy administration (2021; USD per visit)			
OPD IV bolus	Gamma	14	[23]
OPD IV infusion	Gamma	32	Primary data
IPD IV infusion	Gamma	122	[23]
Other healthcare costs (2021; USD per year)			
SD year 1 (OPD regimen)	Gamma	2041	Primary data
SD year 1 (IPD regimen)	Gamma	7321	Primary data
SD year 2	Gamma	2041	Primary data
SD year 3 and subsequent years	Gamma	1875	Primary data
PD year 1 (OPD regimen)	Gamma	3789	Primary data
PD year 1 (IPD regimen)	Gamma	8445	Primary data
PD year 2	Gamma	3789	Primary data
PD year 3 and subsequent years	Gamma	3136	Primary data
*Direct non-medical costs (2021; USD per visit)*			
Food	Gamma	2 (0.4)	[23]
Transportation	Gamma	5 (0.2)	[23]
*Hospital visits rate*			
5-FU/LV (per course)		30	[5]
Capecitabine * (per course)		6	Assumption
FOLFOX (per course)		12	[5]
CAPOX (per course)		8	[5]
FOLFIRI (per course)		12	[5]
SD, latter half of year 1 (off treatment)		6	Primary data
SD, year 2		13	Primary data
SD, year 3 and subsequent years		12	Primary data
PD, latter half of year 1 (off treatment)		9	Primary data
PD, year 2		20	Primary data
PD, year 3 and subsequent years		18	Primary data
***Utilities***			
SD, on IV CMT	Beta	0.600 (0.063)	[16]
SD, on oral CMT	Beta	0.651 (0.047)	[16]
SD, off treatment	Beta	0.850 (0.100)	[16]
PD, on IV CMT	Beta	0.560 (0.101)	[16]
PD, off treatment	Beta	0.624 (0.043)	[16]

* Assumed that patients would have a monthly hospital visit for drug dispensary; CMT, chemotherapy; IV, intravenous administration; IPD, inpatient department; IV, intravenous; OPD, outpatient department; PD, progressive disease; SD, stable disease; USD, US dollars.

**Table 3:** Lifetime costs and health outcomes of each colorectal cancer treatment strategy compared with a base case.

Treatment Options	Total Cost (USD)	LYs	QALYs	Incremental Cost (USD)	Incremental QALYs	ICERs (USD/QALY Gained)	Interpretation
5-FU/LV→FOLFIRI	15,422	4.52	2.14	−1671	−0.40	4214	dominated by 5-FU/LV→CAPOX
5-FU/LV→FOLFOX	17,092	5.31	2.54	-	-	-	base case
5-FU/LV→CAPOX	14,321	5.35	2.56	−2771	0.02	-	cost saving compared to base case
Capecitabine→FOLFIRI	17,719	5.57	2.82	627	0.29	2176	dominated by capecitabine→CAPOX
Capecitabine→FOLFOX	19,477	6.42	3.24	2385	0.71	3377	dominated by capecitabine→CAPOX
Capecitabine→CAPOX	16,532	6.46	3.27	−561	0.73	-	cost saving compared to base case
FOLFOX→FOLFIRI	26,259	6.39	3.37	9166	0.83	10,986	dominated by CAPOX→FOLFIRI
CAPOX→FOLFIRI	21,644	6.87	3.60	4552	1.07	4258	cost effective compared to base case

ICERs, incremental cost-effectiveness ratios; LYs, life years; QALYs, quality-adjusted life years; USD, US dollars.

**Table 4:** Budget impact analysis.

Regimen	Year					Total over 5 Years (USD)	Per Year (USD)
	1	2	3	4	5		
5-FU/LV→FOLFOX *	1,172,438	1,564,064	1,882,169	2,140,896	2,351,667	9,111,234	1,822,247
5-FU/LV→CAPOX	1,172,438	1,716,961	2,159,003	2,518,277	2,810,706	10,377,386,	2,075,477
5-FU/LV→FOLFIRI	1,172,438	1,869,648	2,435,455	2,895,138	3,269,112	11,641,790	2,328,358
Capecitabine→FOLFOX	2,030,097	2,361,654	2,641,650	2,878,404	3,078,891	12,990,697	2,598,139
Capecitabine→CAPOX	2,030,097	2,489,890	2,877,811	3,205,456	3,482,551	14,085,805	2,817,161
Capecitabine→FOLFIRI	2,030,097	2,617,949	3,113,646	3,532,058	3,885,653,	15,179,402	3,035,881
FOLFOX→FOLFIRI	5,805,720	6,337,576	6,797,503	7,195,766	7,541,166	33,677,731	6,735,546
CAPOX→FOLFIRI	4,049,712	4,605,604	5,081,072	5,488,246	5,837,427	25,062,061	5,012,412

* The base case strategy. USD, US dollars.

## Data Availability

The data that support the findings of this study are available from the corresponding author, upon reasonable request. The data are not publicly available due to privacy and ethical reasons.
